# Polyphenols as Antiviral Agents: Their Potential Against a Range of Virus Types

**DOI:** 10.3390/nu17142325

**Published:** 2025-07-16

**Authors:** Nurten Coşkun, Ranya Demir, Ahmet Alperen Canbolat, Sümeyye Sarıtaş, Burcu Pekdemir, Mikhael Bechelany, Sercan Karav

**Affiliations:** 1 Department of Molecular Biology and Genetics, Çanakkale Onsekiz Mart University, Çanakkale 17000, Türkiye; nnurten.coskun@gmail.com (N.C.); ranyaddemir@gmail.com (R.D.); ahmetalperencanbolat@stu.comu.edu.tr (A.A.C.); sumeyyesaritas@stu.comu.edu.tr (S.S.); burcupekdemir0@gmail.com (B.P.); 2 Institut Européen des Membranes (IEM), Centre National de la Recherche Scientifique (CNRS), École Nationale Supérieure de Chimie de Montpellier (ENSCM), Unité Mixte de Recherche (UMR) 5635, University Montpellier, F-34095 Montpellier, France; 3 Functional Materials Group, Gulf University for Science and Technology (GUST), Masjid Al Aqsa Street, Mubarak Al-Abdullah 32093, Kuwait

**Keywords:** polyphenols, antiviral effect, viral diseases

## Abstract

Polyphenols are structurally diverse plant metabolites that have attracted significant interest. Their compositions are versatile, depending on their structures, including the number of rings in the polyphenol composition. Based on these attributes, polyphenols can be classified as flavanols, anthocyanins, flavones, phenolic acids, stilbenes, and lignans. Polyphenols mainly possess inhibition of viral replication, interference with viral protein synthesis, and modulation of immune responses, providing significant antiviral effects against several viruses, including herpes simplex virus, hepatitis C virus, and influenza. They are crucial for medical compounds in diverse, versatile treatments, namely in diabetes, cardiovascular disorders, cancer, and neurodegenerative problems. Plants are the primary source of bioactive molecules, which are valued for their anti-inflammatory, antioxidant, anticancer, and antiviral activities. Especially, polyphenols are extracted as the most abundant bioactive compounds of plants. Moreover, viral infections are one of the major factors in illnesses and diseases, along with bacteria and fungi. Numerous in vitro and in vivo studies report antiviral activity against SARS-CoV-2, Mayaro virus, dengue virus, herpesvirus, and influenza A virus, though clinical validation remains limited. Additionally, inhibition of viral entry, interference with viral replication, modulation of host immune response, and direct virucidal effects were examined.

## 1. Introduction

Polyphenols, which are active substances with over 8000 distinct structures, are recognized as being of great interest [1,2]. Their compositions are versatile, depending on their structure and number of rings, as well as attached groups to these rings [3]. Based on these attributes, the classification of polyphenolic compounds can be made into flavonoids, phenolic acids, stilbenes, and lignans as important components of the polyphenol family [4]. Flavonoids mainly possess inhibition of viral replication, interfere with viral protein synthesis, and modulate immune responses, exhibiting significant antiviral effects against multiple viruses, including hepatitis C virus (HCV), herpes simplex virus (HSV), and influenza [5,6]. Additionally, phenolic acids donate their hydrogen atoms, providing significant anticancer and antioxidant activity. Their activities are significantly crucial for medicinal compounds in multiple, versatile treatments. These treatments can be used in diabetes, cancer, cardiovascular disorders, and neurodegenerative problems [7,8,9,10]. Stilbenes, a type of phenylpropanoid, are a significant class of non-flavonoid phytochemicals [11]. One of the best-known compounds in this category is resveratrol, which is naturally present in grapes and peanuts and abundant in red wine [12]. The composition of resveratrol possesses antioxidant and anti-inflammatory activity, as well as the possibility of preventing chronic diseases [13]. Moreover, lignans are predominantly present in vegetables, legumes, and cereals, suggesting a fiber-rich diet is essential for human health [14]. They significantly exhibit anticancer, anti-inflammatory, antioxidant, anti-menopausal, and antimicrobial activity [15,16]. Plants comprise numerous types of polyphenols, including isovitexin, vitexin, quercetin, diosgenin, rutin, and saponins [17]. Several studies reveal that quercetin possesses antiviral properties against the hepatitis B virus (HBV) [18]. Green tea polyphenols, commonly known as epigallocatechin-3-gallate (EGCG), have potent antiviral activity against various types of hepatitis viruses [19,20].

For centuries, natural bioactive substances have been recognized as potential alternative treatments [21]. These bioactive molecules are primarily sourced by plants, which are valued for their antioxidant/anti-inflammatory, anticancer, and antiviral activities [19,22]. Subsequently, these attributes have gained significant interest as safer options for treatments and new sources of pharmaceuticals [19,23]. Viral infections have been one of the essential topics to study for potential treatment for human health, including hepatocellular carcinoma, type 1 diabetes, and Alzheimer’s disease [24]. During COVID-19, these studies have emerged to comprehend more about viral infections, namely common viral and gastrointestinal infections, including severe acute respiratory syndrome coronavirus 2 (SARS-CoV-2) [25,26]. To explore the nuances of COVID-19 prophylaxis and treatment, alternative bioagents have been investigated, namely, black tea (theaflavins) and green tea (EGCG) polyphenols. Experimental studies revealed that they exhibit significant antiviral activity against single-stranded RNA viruses, key viral proteins (e.g., 3CLpro, RdRp), and receptors (e.g., ACE2) [27]. Additionally, another associated study suggested that polyphenols like quercetin, kaempferol, EGCG, and catechins show efficacy against viruses like influenza and COVID-19 [28,29]. Moreover, a study based on retroviruses, particularly on lentivirus types like human immunodeficiency viruses (HIV), revealed that despite combined antiretroviral therapy (cART) and highly active antiretroviral therapy (HAART) being effective in the treatment of HIV, they were not curative [30]. Therefore, the overall study suggested that natural compounds like flavonoid derivatives, namely, tectorigenin and apigenin, have potential for treatment, especially considering the limitations and side effects associated with combined cART and HAART [31,32]. Another recent study licensed by the Food and Drug Administration (FDA) revealed a list of potential natural products, widely plant-based compounds that are being studied as potential antiviral drugs [33]. According to the analysis, despite an estimated 250,000 higher plant species, only a low percentage have been studied for their potential medicinal properties. This body of research offers a promising avenue to discover novel bioactive compounds. A study that references this analysis aimed to discover and develop unique antiviral drugs with influential antiviral activity, obtained from polyphenolic compounds, including phytochemical antiviral plant extracts, as well as various marine and microbial sources [34]. Despite having several treatments and therapies available, the dengue virus (DENV) infection has been observed on polyphenols such as flavone baicalein, which shows promising potential to address limitations on preventing the DENV infection by exerting a potent activity against the host and post-entry replication [24]. Such marine phytochemicals, including quercetin and narasin, have been observed to exhibit anti-DENV activities [35,36]. Another polyphenol-rich plant, *Aronia melanocarpa* (*A. melanocarpa*), exhibited antiviral activity due to its enriched polyphenolic content as an ethanolic extract [37]. Although these polyphenols possess antiviral activity, their complex mechanisms make it difficult to understand and create limitations to food applications of polyphenols [38,39]. Furthermore, laboratory conditions are very limited for their utility, bioavailability, and antiviral potency [40,41]. To evaluate the bioavailability, several techniques have been investigated, including nanoparticle encapsulation and liposomal technology [22,42]. Depending on the source, storage conditions, and methods of isolation of polyphenols, the required nanoparticle is selected [43,44,45]. Most studies based on antiviral polyphenols are investigated as in vitro or cell line-based experiments. Developmental studies on these compounds require thorough examination to evaluate properly and select a therapeutic dose [19]. In this review article, we have evaluated the general concept of polyphenols and their advantageous effects on health and diseases. Additionally, we evaluated the specific antiviral effects on certain viruses and indicated the mechanisms behind their effects. Relevant studies were also evaluated and summarized in this concept.

## 2. Overview of Polyphenols

(a) Classification of Polyphenols and Sources of Polyphenols: Dietary Sources

**Figure 1:**
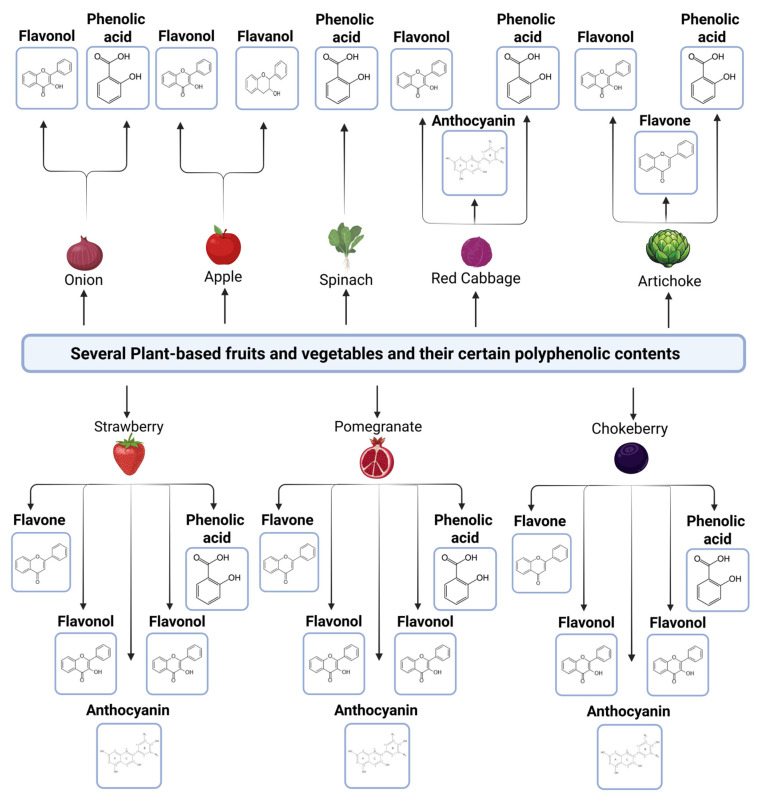
Several plant-based foods with their divergent polyphenolic components.

Polyphenols are plant-based bioactive components of foods, and they can be divergent (Figure 1). Flavonoids are common polyphenols in plants, including onions, tea, grapes, and hot peppers [46]. Several different subclasses of flavonoids contain flavanones, isoflavones, flavonols, flavan-3-ols (catechins), flavones, and anthocyanins [47]. Flavonols are mostly in vegetables like onions, broccoli, and kale, or fruits like berries, apples, and cherries. They have divergent health-promoting effects, including antioxidant, antiviral, and anti-inflammatory effects and activities [48,49]. Additionally, they have common types, particularly quercetin and kaempferol. They are abundant in celery and chamomile, and their common types are luteolin and apigenin, which possess anti-inflammatory and anticancer activities on humans and other living things [50,51]. Flavan-3-ols (catechins) are another subclass of flavonoids [52]. Green tea, berries, and cocoa have epigallocatechin gallate and epicatechin, which are common types of flavan-3-ols. Flavanones, which are sourced from citrus fruits, including oranges and mandarins, are another subclass of flavonoids. The components of citrus fruits provide antiviral and antioxidant effects, and these fruits have flavanones, including hesperidin and naringenin [53,54]. Cyanidin and malvidin are several commonly used examples of anthocyanins that are generally present in maize, berries, red cabbage, and grapes [48,55]. They have anti-inflammatory and antibacterial activity against diseases, as well as the ability to improve cardiovascular health [56,57]. Isoflavones are the last subclass of flavonoids that have several types, including daidzein and genistein, and can provide various effects of polyphenols, namely phytoestrogens, which regulate sexual development, bone health, and anticancer support [58,59].

Phenolic acids are other subgroups of polyphenols that are mostly found in various foods, fruits, and beverages, including tea, coffee, and red fruits [60]. Two of the most frequently detected phenolic acids were found in numerous experiments [61]. The first of these phenolic acids is hydroxybenzoic acid, which is particularly found in tea, onions, and red fruits [62,63]. In addition to these, several common hydroxybenzoic acids are determined to be vanillic acid and gallic acid, which have several health-promoting activities, including antioxidant and antimicrobial [64,65]. Furthermore, another known phenolic acid is hydroxycinnamic acid, which is generally contained in different types of fruits, coffee, and vegetables [66]. Hydroxycinnamic acids can be divided into subgroups, including caffeic, chlorogenic, and ferulic acids, which exhibit anti-inflammatory, neuroprotective, anti-cancer, and antioxidant effects on living organisms [67,68].

The other subgroup of the polyphenols is stilbenes, which are widely detected polyphenols, generally found in grapes, red wine, and berries [69,70]. One such polyphenol is resveratrol, which is used in several medical treatments associated with its longevity-promoting, anticancer, and anti-inflammatory effects [70,71,72]. Other key polyphenols are lignans, which have different types, including pinoresinol and secoisolariciresinol [73,74]. Secoisolariciresinol and pinoresinol are present mostly in flaxseeds, sesame seeds, and whole grains [73,75]. These compounds offer several biological benefits, including phytoestrogenic effects, hormonal balance protection, and antioxidant activity, especially in terms of humans [76,77].

Curcumin and tannins are other common polyphenols; however, they are not in any previously explained subgroups [78]. Curcumin is primarily found in Lakadong turmeric (*Curcuma longa*) and golden milk, and it has several health-benefiting activities, including anti-inhibitory and antiviral effects [79,80]. The other common polyphenols are tannins present in diverse beverages or foods, including red wine, grapes, green tea, black tea, and other fruits, particularly those with red colors [81,82]. They provide antioxidant, antimicrobial, and cardiovascular benefits, which are important for living organisms [83,84].

(b) General Biological Activities of Polyphenols Relevant to Antiviral Activity

Several polyphenol subclasses and types have biological activities associated with antiviral activity [85]. These biological activities can be exemplified by antioxidant, anti-inflammatory, lung-protective, cardiovascular-preservative, cytotoxic, antibiofilm, anticancer, and antibacterial activities [86]. In a study in which curcuminoids and curcumin were obtained from Curcuma longa extract, their potent antiviral, anti-inflammatory, and antioxidant activities were evaluated in SARS-CoV-2-infected human neuroblastoma SH-SY5Y cells [87]. The results demonstrated that plasma membrane-associated transmembrane protease serine 2 (TMPRSS2) and TMPRSS11D expressions were decreased by Me23, which is a curcuminoid. This inhibition positively affected the reactive oxygen species (ROS) level elevated by SARS-CoV-2. Such inhibition showed antioxidant activity associated with antiviral activity. Moreover, Me23 enhanced antioxidative activity by increasing NRF2 gene expression, which has an active pathway in the reduction of pro-inflammatory cytokines, including MCP-1, TNF-a, IL-6e, and IL-1b, while retaining NQO1 activity, which is a mostly abundant enzyme in antioxidative pathways and regulates the expression of the NRF2 gene that deals with the infection. Additionally, Me23 and Me08, which are curcuminoids, effectively reduced the replicative activities associated with the disease resulting from the infection. Furthermore, the anti-inflammatory effects of curcuminoids and curcumin were detected by suppressing the levels of pro-inflammatory cytokines such as IL-6, TNF-α, IL-17, and INF-γ, which can cause demyelination and axonal damage to the cells. Notably, especially Me08, decreased INF-γ levels. These findings demonstrated that curcumin and curcuminoids have anti-inflammatory and antioxidant effects, exhibiting antiviral effects.

In another study, ginger and garlic were combined to detect the bioactive compounds’ enhanced antiviral, antimicrobial, and antioxidant activities, especially polyphenols [88]. Garlic and ginger increased the inhibition of viral and microbial infections and provided each other with activation. Compounds including polyphenols have antioxidant activity, providing antimicrobial and antiviral activity while decreasing the damage from viruses and microbial living. In this study, flavonoid compounds were generally detected, and their activities, which were explained in the study, were investigated. Additionally, researchers showed that the combination of garlic and ginger provided greater antioxidant, antiviral, and antimicrobial properties in these foods than either alone.

In a study determining the antiviral and cytotoxicity activities of quercetin-O-deoxyhexoside, which was obtained from Bauhinia holophylla leaves, the improvement of antiviral and cytotoxicity-determining results was detected [89]. In the antiviral study, Zika virus activity on the African green monkey kidney epithelial cells deals with the cytotoxic effect of the quercetin-O-deoxyhexoside, a phenolic component of the leaves. The results of the study demonstrated that quercetin-O-deoxyhexoside obtained from Bauhinia holophylla leaves was effective while elevating the viral infection mechanism of African green monkey kidney epithelial cells and indirectly altering the cytotoxic activities with an antiviral effect.

According to a study, a polyphenol subclass, chlorogenic acid, served as an increasing factor for antiviral, anti-inflammatory, and antioxidant activities of *Arctium lappa* Linn. [48]. Antiviral activity was determined by the use of the white spot syndrome virus on red swamp crayfish (*Procambarus clarkii*). Moreover, it was attributed to antioxidant, antiviral, and anti-inflammatory activity in various cases, including inhibition of numerous biological pathways.

## 3. Common Antiviral Polyphenols

Despite having versatile benefits, several types of polyphenols have become prominent for their antiviral activities [90,91]. As mentioned, *A. melanocarpa* ethanol extract contains significant polyphenols, namely isoquercetin, kaempferol, caffeic acid, ferulic acid, and hydroxybenzoic acid [92,93]. These polyphenolic constituents demonstrated in vitro and in vivo antiviral efficacy by suppressing influenza infection potential [37]. Another study based on *A. melanocarpa* demonstrated antiviral activity against the human respiratory influenza A virus and human betacoronavirus-1 [94]. A study based on brown alga Ecklonia cava, which is enriched with phlorotannins such as fluorofucofuroecol, phloroglucinol, diecol, ecol, and 7-phloracol, reported that they exhibited antiviral activity against the influenza virus [95]. In the context of green tea polyphenols, they have emerged particularly with EGCG, epigallocatechin, epicatechin, and epicatechin gallate (ECG) [19]. According to a study, EGCG blocks the entry of HCV into cells via viral envelope proteins and inhibits cell-to-cell transmission [20]. Related studies on EGCG determined and explained a potent antiviral activity against the HBV and Zika virus [96,97]. Moreover, EGCG exhibits an antiviral effect against the human herpes virus, and the cytotoxic effect on protein expression and cell viability of the virus was determined [98]. Proceeding with the content, potential applications of EGCG against COVID-19 have also been studied in several studies [99,100]. Recent studies suggested that EGCG has the ability to exhibit an inhibitory activity against the Chikungunya virus by inhibiting the viral infection [101]. In a study based on theaflavins, particularly theaflavin (TF1), theaflavin-3′-monogallate (TF2), and theaflavin-3-3′-digallate (TF3), their responses against HCV in cell cultures were investigated and reported. Experimental results demonstrated that theaflavins block the entry of the virus dose-dependently and showed an inhibitory effect against HCV infection. On the contrary, no activity in HCV replication was expressed when HCV replicon usage [102]. Unlike theaflavins, tannic acid is represented as a potential inhibitor in the early stage of HCV entry due to its ability to block cell-to-cell spreading between cell cultures, and this ability interferes with Huh 7.5 cell cultures [103].

Curcumin is a powder with a yellow-orange color obtained from the root of the *Curcuma longa* plant, which is a polyphenol derivative that has been widely used for centuries in Asian cuisine and as a traditional medicine to treat diseases [104,105]. The antiviral activity of curcumin on HBV infection indicates an inhibitory effect on the expression of HBV by targeting cellular signaling pathways, including Wnt/β-catenin, Ap1, STAT3, MAPK, and NF-κB, which are crucial for virus infection processes. Pomegranate is a fruit that is enriched with polyphenols, significant tannins, and flavonoid compounds [106]. These polyphenols exhibit significant antiviral effects against influenza viruses, including H1N1 and H2N3 strains, by disrupting their structures dose-dependent manner [107]. Experimental studies on *Tilia amurensis* honey express a significant antiviral activity against influenza A virus infection in murine macrophages [108]. Resveratrol, another polyphenolic compound with versatile health-promoting benefits, has been claimed to exhibit antiviral activity against varied members of the Herpesviridae family and rotavirus [109]. Additionally, resveratrol exhibits inhibitory activity against Epstein-Barr virus (EBV) in protein synthesis and viral-induced transcription factors of Burkitt’s lymphoma cells, namely NF-kB and AP-1, affecting individuals [110,111]. According to an investigation that included luteolin, it can suppress the protein expression of EBV-positive cells by preventing virus proliferation. Furthermore, further investigation showed that there was a remarkable reduction in both the number of virus-reactivating cells and viral production [112]. An early study was able to synthesize novel polyphenols, primarily including esters derived from gallic and ferulic acids [113]. The results of the study revealed that they exhibit an inhibitory effect on 12-tetradecanoylphorbol-13-acetate, which triggers the activation of the EBV by promoting infection. One recent study has developed a nanovaccine that was derived from tannic acid and a novel protein antigen to target EBV-induced tumors with interferon-α (IFN-α) or CpG as adjuvants for anti-PD-L1 treatments, which have been studied abundantly in recent years [114]. *Glycyrrhiza uralensis*, also known as Chinese licorice, is enriched with polyphenolic compounds and has various benefits, including anti-arrhythmic properties, which are effective health-promoting treatments [115]. In a study that isolated the polyphenols of this plant, including glyasperin D, licocoumarone, licoflavonol, 2′-methoxyisoliquiritigenin, glyasperin C, and glycyrin, the aim was to investigate their antiviral activity on rotavirus [116]. The results of the investigation demonstrated the antiviral activities of these polyphenolic compounds on rotavirus in vitro, especially in the G5P and G8P groups, by suppressing the replication of rotavirus in the Caco-2 cell line. Another recent in vitro study on Brazilian and Mexican propolis, which have high levels of polyphenolic compounds, particularly rutin, quercetin, and caffeic acid, revealed that they promote antiviral activity against human coronavirus 229E [117]. In addition to these, several polyphenolic activities have also been investigated during COVID-19 infection by studying several polyphenol types, including tea-naringenin, EGCG, herbacetin, eriodicytol, theasinensin-D, resveratrol, oolonghomobisflavan-A, catechin, teaflavin-3-O-gallate, and curcumin [118,119].

In accordance with a 2024 study, the antiviral effect of polyphenol-rich sugarcane extract (PRSE) on the influenza A virus was investigated [120]. The results of the investigation indicated that PRSE suppresses the replication process of the influenza A virus in a dose-dependent manner by reducing viral genome replication, protein expression, and mRNA transcription, without causing any cytotoxicity. During the experiments of the study, identified polyphenols in PRSE composition were listed as chlorogenic acid, luteolin, and tricin. To continue, a review article summarized the incorporation of global health threats of infectious diseases from bacteria and viruses with natural compounds from plants and marine organisms [121]. The summary illustrated the connection between these factors to interpret antibacterial and antiviral properties. According to this interpretation, several names of the antiviral polyphenols were listed, including curcumin, quercetin, and epigallocatechin gallate. Another research article in 2024 was proposed to investigate the polyphenols of the eight most commonly used medicinal plants in Peru. The results of the study identified polyphenols that were listed as rutin, chlorogenic acid, rosmarinic acid, caffeic acid, and gallic acid. To extend the study, these polyphenols were analyzed in silico against the viral proteins ICP27 (Herpes simplex virus-1 (HSV-1)), NS2B/NS3 (dengue virus 2 (DENV-2)), and NS5B (HCV). Results of the analysis revealed that the activity of chlorogenic acid was notable against DENV-2 and HCV, rutin against HCV and HSV-1, and rosmarinic acid against DENV-2 and HCV [122]. Proceeding with the content, polyphenolic compounds from *Maackia amurensis* heartwood are recently being studied for their neuroprotective and antiviral potential against herpes simplex virus type 1 (HSV-1). The results of the study indicated that these compounds were able to suppress ROS levels and enhance mitochondrial function. Further investigation of this study indicated that several of these polyphenols, primarily maackin and scirpusin A, exhibited the highest anti-HSV-1 activity [123].

Another research study aimed to evaluate *Cistus laurifolius* (*C. laurifolius*) and to determine its polyphenolic effects, which is widely used for several skin disease treatments in Turkey [124]. The study intended to develop an herbal lip balm to investigate polyphenolic compounds of *C. laurifolius* against HSV-1. Clinical studies of this investigation revealed that the herbal balm that is enriched with the polyphenolic compounds of *C. laurifolius* exhibits significant antiviral activity against HSV-1. In accordance with Zima, Katarzyna et al., a polyphenol-rich blend that consisted of *Echinacea purpurea*, *Lonicera caerulea* var. *kamtschatica sevast*., and *A. melanocarpa* was tested against human coronavirus OC43 (HCoV-OC43) [125]. Results of the study revealed that the blend exhibited promising antiviral activity with immunomodulatory and antioxidant effects by affecting the viral replication cycle of HCoV-OC43 by blocking viral entry to the host cells.

## 4. Mechanisms of Antiviral Action of Polyphenols Associated with Several Common Viruses

**Figure 2:**
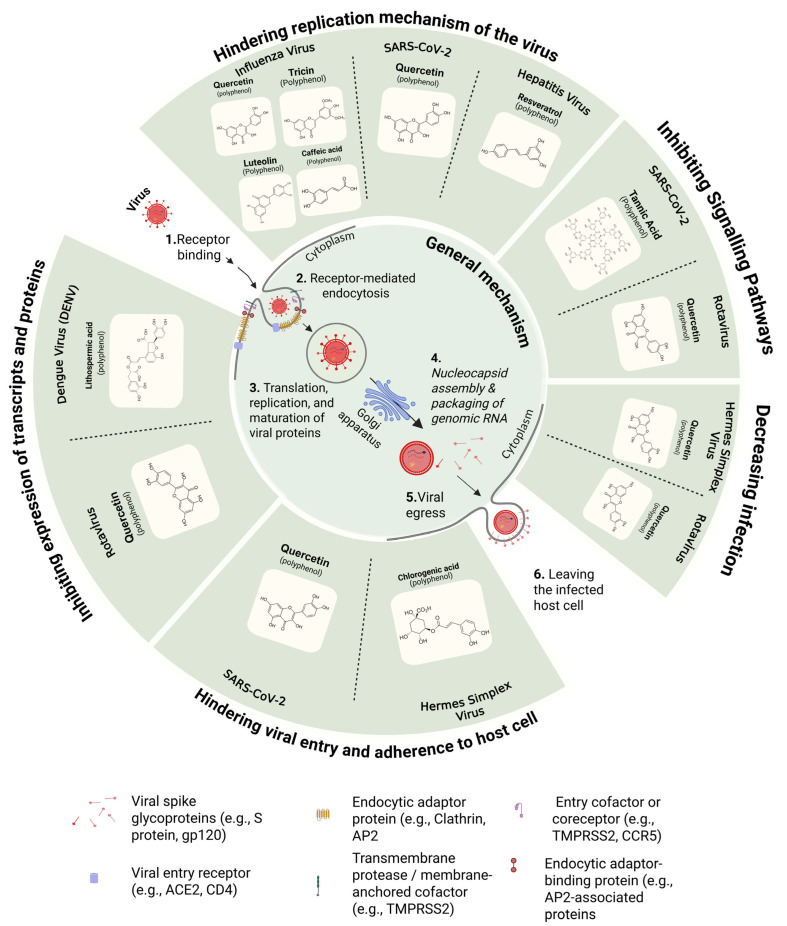
General summary of polyphenols and their multi-targeted antiviral strategies to different types of viruses.

Viral infections are major factors in illnesses and diseases, along with bacteria and fungi [126,127,128,129]. There have been a complete set of evidenced antiviral effects of various phenolic compounds on diseases including SARS-CoV-2, Mayaro virus, dengue virus, Newcastle disease virus, Murine norovirus, Feline Herpesvirus type-1, Human Herpesvirus type-1, influenza A virus, Mouse coronavirus MHV-A59, white spot syndrome virus, and Chikungunya viral infection (Figure 2) [2,125,130,131]. SARS-CoV-2 pseudo-virions were also investigated with polyphenols and evidenced that viral particles can also be affected by these compounds [132].

(i) SARS-CoV-2

During the 2019 pandemic, the SARS-CoV-2 virus caused a huge global epidemic and impacted the entire planet. Studies based on SARS-CoV-2 brought remarkable results and gave rise to the importance of polyphenolic compounds [133,134]. In a newly published study, it has been proposed to identify triphenol compounds and their potential to help the inhibition capability of these phenols on different compartments of the virus, including SARS-CoV-2 spike protein receptor binding domain (S-RBD), SARS-CoV-2 3-chymotrypsin-like protease (3CL^pro^), and angiotensin-converting enzyme 2 (ACE2) [47]. The study demonstrated that bioactive polyphenols, namely benserazide hydrochloride and exifone, inhibited the activity of SARS-CoV-2 3CLpro protease, a preserved cysteine protease that is vital for the coronavirus replication processes [134]. Specifically, benserazide hydrochloride exhibited time-dependent inhibition effects, whereas exifone did not have any significant results. Correspondingly, the mode of inhibitory activities of these triphenols with 3CLpro was further investigated. According to this further investigation, these two triphenols were exhibiting inhibitory activities where exifone binding at a cleft between domains II and III of 3CLpro through hydrogen bonding, and benserazide hydrochloride interacting in the catalytic pocket of this enzyme. The results also pointed out that exifone exhibited antiviral activity against particularly several types of SARS-CoV-2 pseudovirus strains dose-dependently with the inhibition of viral entry into hACE2-HEK293 T cells that are important for viral infection. It was also demonstrated that exifone impeded the association between ACE2 and S-RBD, which is a critical pathway in the viral infection of human cells [135].

There are various polyphenols whose effects on SARS-CoV-2 have been studied extensively [136]. Tannic acid is a polyphenol found abundantly in cereals, tea, vegetables, red wine, herbs, and coffee [137]. The therapeutic properties of this phenolic compound have been studied, and it has been exposed that tannic acid is showing promise as a therapeutic agent [138,139]. In a dedicated study on tannic acid, the host-protein interactions, anti-inflammatory, and antioxidant efficiency of this compound were investigated [127]. The in silico analysis proved that tannic acid possesses interaction capability with key virulence factors such as TLR-4, MAPK, COX-2, and NF-κB, playing key roles in the inflammatory responses and immune modulation during viral infections in the immunopathogenesis of SARS-CoV-2. Furthermore, tannic acid is capable of decreasing and partially preventing MAPK and NF-κB signaling pathways, which are present in viral infection, by hindering mediator production, leading to the alleviation of organism response [140,141]. The results also reported that the tannic acid possesses a binding affinity to COX-2, suggesting the ability to bind SARS-CoV-2 proteins, taking a role in viral entry and replication, strongly and with low energy requirements. In a dedicated study examining the ex vivo ability of quercetin in the decrease of SARS-CoV-2 replication, the mechanism behind this function has been elucidated [136]. By infecting green monkey kidney Vero E6 cells and human colon carcinoma Caco-2 cells and incubating them in the quercetin media, the replicated viral RNA was measured by the RT-qPCR technique. The obtained results indicated that quercetin successfully inhibited SARS-CoV-2 replication in both cells in a concentration-dependent manner. With the aid of decreased expression levels, spike and ACE2 co-expressions were also decreased. The mechanism behind this inhibition indicated that quercetin prevents the syncytium formation mechanistically and facilitates virion propagation. After data collection, results have been assessed that quercetin has the potential to be used as a therapeutic agent against COVID-19.

(ii) Influenza Virus

Influenza infections are the most common type of viral illnesses, which are seasonal, antigenically variable, and generally impact the populations annually [125]. The effects of influenza viruses on the respiratory system affect a wide range of age groups and may greatly impact the mortality of elderly and chronically ill patients [142]. Polyphenols have been studied on influenza viruses, and their antiviral effects have been reported [120]. In a recent study, it was reported that polyphenol-rich sugarcane extract (PRSE) was found to be antiviral against a wide class of influenza A virus (IAV) strains in vitro [120]. IAV is an enveloped and negative-sense ssRNA virus in the Orthomyxoviridae family [143]. As a result, PRSE was shown to exhibit inhibitory properties on a wide range of IAV strains, mainly H1N1 and H3N2 subtypes, in vitro. The liquid-chromatography-mass spectrometry (LC-MS) results revealed that chlorogenic acid, a polyphenol found in the polyphenol composition of PRSE along with tricin and luteonin, exerted the antiviral activity of this extract and potentially inhibited the neuraminidase activity [120,144]. It was also reported that this extract was potentially targeting the early replication stage of IAV replication. Therefore, PRSE was concluded to be an antiviral agent candidate, yet further and specific studies were advised in the elucidation of the mode of action and direct target of PRSE. The prevalence and variety of phenolic compounds indicate that diverse plant species can be exploited as polyphenol sources, and their effects can be identified to assign potential pharmaceutical components. Apiaceae plants are widely used in studies owing to their various pharmacological benefits, namely anti-inflammatory, anti-cancer, antimicrobial, and antioxidant properties, as well as other remarkable effects (hypoglycemic, anxiolytic, and so on). Since their antiviral effects have not been investigated extensively, Apiaceae plants, and specifically *Peucedanum japonicum* (also known as coastal hog fennel or Sacna), have been used in a dedicated study to elucidate and identify their active components taking a role in the function of their constituents along with antiviral properties. This plant is especially common in Far East countries such as Japan, South Korea, and China and has been used traditionally to treat cough diseases. The antiviral effects of the plant were studied on influenza virus strains that exhibit resistance to current traditional drugs. After carefully examining experiments, the results showed that all Apiaceae plants successfully inhibited influenza viruses. Sacna, on the other hand, hindered the replication mechanism of influenza strains. The insight of the inhibition pointed out the multiplication of A-type viruses, H3N2 and H1N1, and B-type viruses, oseltamivir-resistant and amantadine-resistant, were blocked by Sacna extract polyphenols. Additionally, the viral replication phase was also inhibited by Sacna. After the mode of action was verified, the antiviral compounds were identified by the LC20ADXR high performance liquid chromatography system. The bioactive components were found as flavonoids namely luteolin and quercetin, and other classes of polyphenols, namely umbelliferone, hymecromone, and caffeic acid. The whole result concluded that caffeic acid was the key compound for the antiviral activity of Sacna plants. This study is valuable since the anti-influenza activity of Sacna plants was first reported here, according to the authors, leading to the potential of this plant as a novel candidate in the therapy of influenza virus types.

(iii) Hepatitis Virus

Hepatitis emerging from viral infections has been a devastating health problem worldwide, affecting millions of people every year [145,146]. The severity and mortality of hepatitis viruses are remarkably high, and the infection pathways vary depending on the virus belonging to the hepatitis family [140]. It is estimated that hepatocellular carcinoma, one of the most common cancer types, is associated with the hepatitis family, mainly with B and C viruses. Several affiliated investigations have been designed to examine the activities of numerous polyphenols on the mechanism of these viruses. In a study focused on HBV, the effects of resveratrol were investigated [147]. RES is capable of exhibiting antioxidant and antiviral properties. HBV is known to raise oxidation, ultimately causing liver injuries. The main purpose was to examine the positive effects of resveratrol on the oxidative stress resulting from oxidation pathways, specifically SIRT1-Nrf2 regulation, and the replication of B-type viruses. The study concluded that resveratrol enhanced the viability of cells and modulated SIRT1-Nrf2 regulation by upregulating SIRT1, promoting Nrf2 phosphorylation pathways, and providing antioxidative actions. The ultimate result revealed resveratrol hindered the replication of HBV. The responsible protein, HBc, and HBV DNA were hindered successfully by resveratrol, concluding this phenolic compound is a promising candidate in HBV-directed liver infections.

(iv) Herpes Simplex Virus

HSV is a widespread human pathogen, with HSV-1 primarily affecting the orofacial mucosa and HSV-2 targeting the genital mucosal surfaces (HSV-2) [148]. During active infection, the virus leads to the development of vesicular lesions in the epithelial tissues. It subsequently spreads to sensory neurons, where it establishes a lifelong latent infection. In efforts to better understand HSV pathogenesis and explore new therapeutic approaches, considerable attention has been given to biologically active compounds—particularly polyphenols—due to their proven antiviral properties [126,148]. In a recent study, Ajwa date extract was investigated to determine its phenolic profile and the potential effects of these phenolic compounds [126]. Accordingly, 17 bioactive compounds with their anti-HSV effects were detected, including flavonoids and phenolic acid derivatives. These identified phenolic compounds were able to exhibit significant responses against HSV type I by hindering viral cell adherence to protect the host cell. One of these phenolic compounds, chlorogenic acid, which is within the composition of Ajwa extract, was found to be the most active component by binding to glycoprotein D and blocking the viral entry mechanisms. Except for specific plant extracts, common types of polyphenols have also been investigated. In a recent study, common polyphenols, namely quercetin, resveratrol, acyclovir, and doxorubicin, were examined on HSV-1 strains. Experimental studies indicated that quercetin successfully decreased HSV-1 infection in a dose-dependent manner. Furthermore, inflammation occurring during viral infections was reduced by quercetin, concluding that it has a potential in the treatment of side effects of HSV-1 infections. Doxorubicin, identified as an anticancer drug, successfully inhibited HSV-1 infection at lower doses than quercetin as well as acyclovir, showing significant changes in viral titrations. Interestingly, no significant effect was found for resveratrol.

(v) Dengue Virus (DENV)

The dengue virus is a harmful virus of the Flaviviridae family composed of a single-stranded RNA structure [149]. Structurally, its virion possesses three structural proteins, namely membrane, envelope, and core, along with non-structural proteins. Envelope proteins are responsible for the biological organization of this virus, as they interact with receptors found on the host cells and infect the organism [149]. *Lithospermum erythrorhizon* is known as one of the commonly used medicinal plants in China and has been used in various clinical studies [140]. The study mainly focused on the active components of plant extracts, including ethanol and lithospermic acid. Results of the study revealed that both extracts exhibited an inhibitory effect on viral replication of dengue virus. Furthermore, *Lithospermum erythrorhizon* has been widely used for a long time for the treatment of severe effects of various diseases, including cancer, viral infections, inflammation, and rheumatism. Lithospermic acid extract has the capability to exhibit effective antiviral activity in the beginning of the replication. This response of the plant extract is represented as an interference with the viral proteins E and NS3 expression pathways. Moreover, lithospermic acid was reported to express an indirect antiviral effect by binding protein E and inhibiting viral activities. Correspondingly, the overall result of these studies summarizes that this phenolic compound is exhibiting effective viral responses against dengue virus infections and can provide insights for therapeutic applications.

Every type of therapeutic plant or herb is commonly used to examine its effects on a specific diseases or illnesses. *Arachis hypogaea* is a biologically beneficial plant that has been employed in a dedicated study for DENV [150]. The study aimed to examine the antiviral and cytotoxicity of ethanol extracts from this plant to combat DENV-2. As a result, tegument ethanolic extract (TEE) of *A. hypogaea* completely stopped the DENV-2 infection by hindering viral entry mechanisms and intracellular organisms. Moreover, specific virions of DENV-2 were also detected by TEE. Although the seed ethanolic extract of *A. hypogaea* was also obtained, there was no significant effect compared to TEE. This study ultimately reported that the pure compounds in TEE should be investigated to address the direct antiviral components and mechanisms of TEE extracts on any dengue virus.

(vi) Rotavirus

Rotavirus is a non-enveloped double-stranded RNA (dsRNA) responsible for infectious diarrhea specifically in infants and children [151]. The structural and non-structural abundance of viral proteins gives rotaviruses their complexity and identity elements such as host specificity, the entry mechanism, and enzymatic reactions for the production of viral transcripts [151]. According to the FDA, there is no approved drug or therapy, remaining an important problem currently. The inquiry into the active component in challenging rotavirus leads to various studies designed with various sources, including polyphenols. In a dedicated study, it was indicated that quercetin, a natural flavonoid, has potent anti-rotaviral effects. In vitro, it significantly reduced viral replication and protein expression in simian, bovine, and human RV strains. In vivo, quercetin-treated mice showed lower viral titers and reduced viral protein levels in the small intestine. The antiviral effect was interferon-independent and associated with the inhibition of RV-induced early NF-κB activation. Overall, the study highlights quercetin’s potential as a therapeutic agent against rotaviral diarrhea.

Table 1 demonstrates the antiviral effect of diverse polyphenols on the different models, and it also shows the mechanisms of antiviral action. However, while in vitro studies demonstrate the antiviral activity of polyphenols at certain concentrations (e.g., 20–50 µM), translating these treatment concentrations into human therapy requires consideration of pharmacokinetics. Most dietary polyphenols have low bioavailability, and such treatment concentrations are generally not fully acceptable through normal consumption. Therefore, the doses used in cell culture studies may not be realistic for human application without chemical modification, novel delivery systems, or intravenous administration. Furthermore, we need several types of evidence before we can accept polyphenols as therapeutic agents for viral infections in humans or animals. This includes well-designed preclinical studies to detect efficacy and safety, followed by randomized controlled clinical trials in humans. Moreover, detailed pharmacokinetic and bioavailability data are essential to ensure that effective concentrations can be achieved in target tissues or organs. Pharmacokinetics is an branch of science that examines the intake, absorption, distribution, metabolism, and excretion of a drug or substance according to their treatment concentration. Without this evidence, it remains uncertain whether polyphenols can move from promising laboratory findings to viable clinical treatments.

## 5. Conclusions

Polyphenols are plant-derived bioactive compounds that exhibit several health-promoting activities, and they have divergent subclasses that have distinct activities, including antioxidant, anti-inflammatory, and antiviral. They can also prevent several diseases, especially viral infections. When the studies are evaluated, it has been determined that polyphenols obtained from different sources exhibit various effects against various viral infections due to their potent antioxidant and antiviral activities. It has been demonstrated that polyphenols can play an effective role in inhibiting viral entry, suppressing viral replication, and reducing viral spread in infections including herpes simplex virus, rotavirus, dengue virus, SARS-CoV-2, hepatitis virus, and influenza, which have recently been responsible for a substantial number of illnesses. Divergent specific viruses are affected by several specific polyphenol types, including catechin, epigallocatechin-3-gallate, curcumin, resveratrol, and quercetin. For instance, catechin, curcumin derivatives, and quercetin are effective on influenza A virus infections, while resveratrol exhibits antiviral activity by hindering expression and signaling pathways of hepatitis B virus. Furthermore, biological compounds such as lithospheric acid tend to exhibit antiviral activity against dengue virus by suppressing expression pathways of viral proteins like E and NS3. Moreover, flavonoids like quercetin show a potent inhibitory activity against rotavirus by reducing viral replication and protein expression. Additionally, SARS-CoV-2 infection is affected by hymecromone, brazilin, curcumin, and resveratrol polyphenols. Accordingly, pioneering studies have been proposed to discover the potential of polyphenols in preventing these diseases and contributing to the development of treatments. While several studies have explored the antiviral effects of polyphenol-rich products, including green tea, berries, and other plant-based foods, the efficacy and potential side effects of many polyphenols have not yet been fully elucidated. Moreover, the antiviral properties of polyphenols vary depending on their source and specific type. Considering these factors, it is evident that further research and clinical studies are required. Inferences in this review showed the antiviral effects of polyphenols; however, most current findings deal with in vitro and animal experiments. As a result, the evidence is limited in the direct usage of antiviral polyphenols on humans. Future studies should determine suitable doses and usage of polyphenols, especially in humans. Additionally, the new studies to be conducted on herpes simplex virus, dengue virus, rotavirus, and other viruses are required for more detailed research. Although in vitro studies show antiviral activity of polyphenols at certain concentrations (e.g., 20–50 µM), translating these findings into human therapy requires careful pharmacokinetic evaluation. Due to their low bioavailability, such concentrations of polyphenols are often unrealistic through normal dietary intake, including oral administration. Thus, without advanced delivery systems or chemical modifications, these treatment concentrations may not be clinically realistic, particularly for humans. To consider polyphenols as antiviral treatments, robust preclinical studies and randomized clinical trials are essential, along with detailed pharmacokinetic and bioavailability data.

## Figures and Tables

**Table 1:** Studies of polyphenols against viral infections in vitro.

Virus	Polyphenols	Model	Mechanisms of Antiviral Action	Treatment Concentration	Ref.
Hepatitis B virus	Curcumin	In vitro (HepG22.15 and Huh-7)	Triggers a cell-type-specific response in hepatoma cell lines and prevents an adaptive cellular optimization that enhances replication of the hepatitis B virus.	20 µM for 72 h	[91]
	Catechin/Epicatechin	In vitro (HepG22.15)	Particularly inhibit the viral antigen surface and show antiviral effect.	50 µM for 5 days	[145]
	Polyphenol-rich *Ilex paraguariensis* extract (quercetin, kaempferol, rutin, caffeic acid, chlorogenic acid)	In vitro (HepG22.15)	Its antiviral phenolic compounds exhibit potential therapeutic efficacy.	10 µg/mL	[152]
SARS-CoV-2	Exifone and benserazide hydrochloride	In vitro (protein-based assays: 3CLpro inhibition, ACE2-S-RBD interaction)	Impede the 3CLpro protease activity vital for SARS-CoV-2 replication.	IC_50_: (exifone: 3.18 µM; benserazide hydrochloride: 0.37 µM)	[47]
	Tannic acid	In vitro, in silico, in vivo (*Danio rerio*)	Prevents the virus uptake to cells by regulating the proteins and exhibits an antioxidant role in ROS that is caused by viral infection.	50 μg/mL	[127]
	Curcumin-containing film spray	In vitro (Vero and MDCK cells)	Inhibit inflammation and apoptosis in alveolar epithelial cells, adjust macrophage polarization, and protect alveolar epithelial cell integrity.	EC_50_: 3.15 µg/mL	[78]
	*Abies* *sachalinensis* (kaempferol, quercetin derivatives, ferulic acid, p-coumaric acid, lignans)	In vitro (African green monkey kidney cells: Vero)	Exhibits an inhibitory effect on the viral infection.	Original extract (undiluted)/1 min	[133]
	Brazilin and theaflavin-3,3′-digallate	In vitro (human alveolar epithelial cell line A549)	Exhibits multiple anti-SARS-CoV-2 activities.	25 μg/mL	[132]
Influenza	Polydatin	In vitro (Vero E6 African green monkey kidney cells, LGC, and MDCK Madin-Darby canine kidney cells)	Its treatment reduces IL-6 cytokine production by correcting its anti-inflammatory properties during the influenza A virus infection.	40 µg/mL	[29]
	*Peucedanum japonicum* (Sacna extract: quercetin, luteolin, caffeic acid)	In vitro (Madin–Darby canine kidney cell line: MDCK)	Inhibits the viral replication of both types of influenza A and B infection.	2 mg/mL	[38]
	Curcumin-containing film spray	In vitro (Vero and MDCK cells)	Inhibit inflammation and apoptosis in alveolar epithelial cells, adjust macrophage polarization, and protect alveolar epithelial cell integrity.	EC_50_: 6.32 µg/mL (influenza B); 7.24 µg/mL (influenza A/H1N1); 12.5 µg/mL (influenza A/H3N2)	[78]
	Polyphenol-rich *Spiraea* extracts (chlorogenic, gentisic, caffeic, ferulic and cinnamic acids, quercetin, quercitrin, luteolin-7-glucoside)	In vitro (Madin–Darby canine kidney cell line: MDCK)	Shows a highly antiviral effect on the influenza A virus (H1N1) by blocking replication.	5.9 µg/mL	[129]
	Polyphenol-rich sugarcane extract (caffeic acid, chlorogenic acid, ferulic acid, p-coumaric acid, sinapic acid, apigenin, luteolin, tricin, quercetin, rutin, catechin, epicatechin)	In vitro (Madin–Darby canine kidney cell line: MDCK)	Blocks the H3N2 and H1N1 replication.	IC_50_: 0.45 mg/mL	[120]
Dengue virus	Lithospermic acid	In vitro (Vero: African green monkey kidney cells)	Inhibits viral replication by binding envelope protein and Non-Structural Protein 3 which are important for viral uptake, at the onset of infection.	EC_50_: 6.50 μg/mL	[140]
	Catechin	In vitro (human hepatoma cells: (Huh 7); (human lymphoblast cells: K562); (baby hamster kidney: BHK-21); (*Aedes albopictus* larvae cells: C6/36)	Inhibits dengue virus replication.	IC_50_: 6.422 µM	[153]
	*Arachis hypogaea* L. extract (resveratrol, caffeic acid, ferulic acid, quercetin, catechin)	In vitro (African green monkey kidney cells: Vero)	Acts in the viral adsorption–penetration stage and inhibits the first steps of infection in the post-penetration stage.	IC_50_: 3.47 μg/mL	[150]
	Cranberry pomace extract (cyanidin, quercetin, myricetin, kaempfer)	In vitro (human lung carcinoma A549 cells); (human hepatoma (Huh 7.5 cells) and in vivo (*Danio rerio*)	Blocks viral entry by preventing viral attachment to host cells.	25–2000 µg/mL for A549 and Huh 7.5 cells; up to 2000 µg/mL for zebrafish	[154]
Herpes Simplex Virus Type 1	Quercetin	In vitro (African green monkey kidney cells: Vero)	Reduce viral infectivity and show significant potential for virus suppression.	62–125 µM	[148]
	Ajwa date extract (gallic acid, ferulic acid, caffeic acid, quercetin, kaempferol, catechin, epicatechin)	In vitro (African green monkey kidney cells: Vero)	Protects cells by preventing virus uptake into host cells.	IC_50_: 113.99 μg/mL	[126]
	*Kalanchoe daigremontiana* extract (gallic, chlorogenic, ferulic, caffeic, and p-coumaric acids)	In vitro (African green monkey kidney cells: Vero); (human HaCaT keratinocytes)	Blocks virus attachment, penetration, and infection.	0.16 g/mL	[1]
Zika virus	Cranberry pomace extract (gallic acid, caffeic acid, quercetin, cyanidin)	In vitro (human lung epithelial A549 cells); (human-derived Huh-7.5 hepatoma cells)	Acts on viral particles and thus prevents their adhesion to the cell surface, being a potential inhibitor of virus entry into the host cell.	26 µg/mL	[154]
Rotavirus	*Opuntia ficus-indica* peel (gallic acid, caffeic acid, chlorogenic acid, ferulic acid, p-coumaric acid, quercetin)	In vitro human breast cancer cells (MCF-17)	Anti-proliferative activity and significant reduction in cell viability	400 µg/mL	[155]
Newcastle disease virus	*Pongamia pinnata* L. seed-derived karanjin	In vitro (chicken embryo fibroblast cells: DF-1)	Enhances antiviral responses and influences glucose metabolism. Reduces virus replication.	3.125–25 μM	[6]
Human Papillomavirus	Epigallocatechin-3-Gallate	In vitro (human foreskin keratinocytes: HFK- HPV18)	Shows anti-viral activity by targeting the E6 and E7 proteins.	100–150 µM	[21]
Mayaro virus	Epigallocatechin-3-Gallate	In vitro (baby hamster kidney: BHK-21	Shows antiviral activity against Mayaro virus by targeting its replicative cycle.	8.3–25 µg/mL	[90]
Murine norovirus	*Polygonum aviculare* extract (quercetin, kaempferol, rutin, gallic acid, caffeic acid, ferulic acid)	In vitro (RAW 264.7 cells) and In situ (cabbage surface inoculated with MNV-1)	Efficiently inactivates norovirus and prevents the infection.	IC_50_ = 78.4 µg/mL	[156]
Mouse coronavirus MHV-A59	P2Et and anamu SC extracts from *Caesalpinia spinosa* and *Petiveria alliacea* (tannins, gallic acid derivatives, ellagic acid)	In vitro (B16–F10 murine melanoma cell line)	Exposure of calreticulin on the surface, which is induced during infection.	IC_50_: 119.6 μg/mL (P2Et extract); 226 μg/mL (anamu SC extract)	[2]
